# The characterization of microbial communities and associations in karst tiankeng

**DOI:** 10.3389/fmicb.2022.1002198

**Published:** 2022-10-19

**Authors:** Cong Jiang, Yuanmeng Liu, Hui Li, Sufeng Zhu, Xiang Sun, Kexing Wu, Wei Shui

**Affiliations:** ^1^College of Urban and Environmental Sciences, Peking University, Beijing, China; ^2^College of Environment and Safety Engineering, Fuzhou University, Fuzhou, China; ^3^Ecology and Nature Conservation Institute, Chinese Academy of Forestry, Beijing, China

**Keywords:** unique habitat, co-occurrence network, amplicon sequencing, karst tiankeng, microbiome

## Abstract

The karst tiankeng is a special and grand negative terrain on the surface, that maintains a unique ecosystem. However, knowledge about bacterial and fungal communities in karst tiankengs is still limited. Therefore, soil samples from five karst tiankengs were collected and subjected to high-throughput sequencing of 16S rRNA and ITS genes, and multivariate statistical analysis. The results showed abundant and diversified bacterial and fungal communities in karst tiankeng. The bacterial communities were dominated by *Proteobacteria* and *Acidobacteria*, and the fungal communities were dominated by *Ascomycota* and *Basidiomycota*. Statistical analysis revealed significant differences in bacterial and fungal communities among the five karst tiankengs, which may indicate that the distribution of bacterial and fungal communities was driven by separate karst tiankengs. The co-occurrence network structure was characterized by highly modularized assembly patterns and more positive interactions. The keystone taxa were mainly involved in nutrient cycling and energy metabolism. The null model analysis results showed that the stochastic process, especially dispersal limitation, tended to be more important in controlling the development of bacterial and fungal communities in karst tiankeng. The bacterial community structure was significantly associated with soil properties (SWC, TN, AN, and BD), while the fungal community structure was significantly associated with soil properties (SWC and TP) and plant diversity. These results can expand our knowledge of the karst tiankeng microbiome.

## Introduction

Carbonate rocks are the material basis for the development of karst landforms, with a total distribution area of 3.44 million km^2^ in China, accounting for approximately 1/3 of the country’s land area (Yuan, 2005). China has the largest karst region in the world (Zhu and Chen, 2005). The karst tiankeng is a typical karst landscape that develops in specific karst geological, geomorphological, climatic, and hydrological environments (Zhu and Waltham, 2005; Shui et al., 2015). The latest research defines karst tiankengs as karst closed pits with a width and depth of more than 100 m, a small ratio of diameter to depth, a continuous circumference, and vertical or subvertical walls (Gunn, 2019). It can be seen that karst tiankeng is a large-scale negative topographic geological wonder on the surface. China is the karst tiankeng kingdom, accounting for more than 70% of the total number of karst tiankengs (Pu et al., 2017). Karst tiankeng tends to appear in groups and form complex cave-hydrogeological systems with underground rivers and caves (Xu et al., 2009; Shui et al., 2015).

Because karst tiankengs are characterized deep into the surface and cliffs, the internal habitat of karst tiankengs is relatively independent of the land surface environment, with unique hydrothermal conditions and primitive microclimates (Zhu and Chen, 2005; Pu et al., 2019). The unique habitat of karst tiankengs serves as an environment for an abundant and unique resource of animals, plants, and microorganisms (Batori et al., 2017; Su et al., 2017; Jian et al., 2018; Jiang et al., 2022a). Karst ecosystems are well known to be characterized by soil erosion, poor soil nutrients, and biodiversity loss (Clements et al., 2006). The karst tiankeng are similar to “oases” in degraded landscapes and play an important role in karst ecosystems. Existing studies have demonstrated that karst tiankengs are important repositories for plant diversity conservation (Su et al., 2017; Chen et al., 2018b; Shui et al., 2022). As the engine of biogeochemical cycles, the role of soil microorganisms in karst tiankengs cannot be ignored (Balser and Firestone, 2005; Fierer, 2017). Meta-analyses have shown that microbial communities are significantly affected by the habitat environment (Delmont et al., 2011; Wang et al., 2013). Due to their nutrient-rich conditions, microbial communities thrive in karst tiankengs, with higher alpha diversity of microbial communities than land surface habitats (Pu et al., 2019; Jiang et al., 2021). The DSE (dark septate endophyte) resources in Dashiwei karst tiankeng were abundant and some of them possess positive effects on plant growth (Lan et al., 2017). In addition, karst tiankengs resemble a natural open top chamber (OTC) and are ideal for studying the response of terrestrial ecosystems, especially fragile karst ecosystems, to climate warming (Yang et al., 2019). Climate warming will alter soil microbial community structure and activities, which is critical to ecosystem functioning and stability (Yuan et al., 2021). The study of microbial communities in karst tiankeng ecosystems can deepen our understanding of global microbial diversity.

The preliminary study of microorganisms in karst tiankeng focused on the macrofungal species and plant fungi (Deng and Wu, 2014; Lan et al., 2017). Jiang et al. (2014) isolated strains with high keratinase yield in karst tiankeng soil through traditional culture techniques, which had the best effect on feather protein degradation. With the advent of high-throughput sequencing technology, the soil microbial community structure and functionality in karst tiankengs have been gradually discovered (Pu et al., 2019; Jiang et al., 2022a,b). The soil microbial communities of karst tiankeng exhibit significant habitat heterogeneity. However, these studies tend to be limited to characterizing soil microbial communities in karst tiankeng, ignoring the interactions of karst tiankeng microbes. In isolated karst tiankeng ecosystems, microbial community survival activity and interactions are critical to ecosystem stability. However, our understanding of the characterization of the microbiome and interactions of karst tiankeng remains poorly understood. Molecular ecological network (MEN) models based on stochastic matrix theory (RMT) can better simulate the interaction between different species in the community (Deng et al., 2012; Yuan et al., 2021). Microbial interactions constrain their ecological functions *via* competition, syntrophism, or symbiosis (He et al., 2017; de Vries et al., 2018). The abundant soil nutrients, plant cover, and unique microclimates may distinguish the microbial trophic structures of karst tiankeng ecosystems from those of general terrestrial ecosystems. Understanding how microbial co-occurrence patterns occur in separate karst tiankeng ecosystems is urgently needed.

In this study, soil samples were collected from five karst tiankengs in the Zhanyi karst tiankeng group and subjected to high-throughput sequencing of bacterial 16S rRNA and fungal ITS1 genes and multivariate statistical analysis. The main purposes of this study were: (i) to determine the taxonomic composition and structure of microbial communities in karst tiankeng, (ii) to evaluate the microbial co-occurrence patterns and assembly processes of bacterial and fungal communities in karst tiankeng, and (iii) to determine the key factors driving bacterial and fungal communities in karst tiankeng.

## Materials and methods

### Sample collection and measurement

We carried out this study in 2021 in Zhanyi District, Qujing City, Yunnan Province, China; the study was performed at Haifeng Natural Reserve (25°35′-25°57′N, 103°29′-103°39′E). The Zhanyi karst tiankeng group includes dozens of karst tiankengs of varying sizes. The annual precipitation ranges from 1073.5 to 1089.7 mm and is affected by the subtropical highland monsoon climate. The annual temperatures ranges from 13.8 to 14.0°C. The soil type was Yunnan red soil.

Among the Zhanyi karst tiankeng group, we selected five karst tiankengs, including Bajiaxiantang (BJXT), Shaojiaxiantang (SJXT), Shenxiantang (SXT), Wangjiaxiantang (WJXT), and Xiaotiankeng (XTK; Supplementary Figure 1). All five karst tiankengs are scattered in inaccessible areas, and only local residents occasionally enter these karst tiankengs, so they are largely kept in pristine conditions. The morphological characteristics of these five karst tiankengs are listed in Supplementary Table 1. Since the XTK slope is a vertical cliff, the seven sampling sites are all located at the bottom of the karst tiankeng. Of the other four karst tiankengs, the eight sampling sites include two at the bottom of the tiankeng and six at the slope of the tiankeng. Each sampling (10 × 10 m^2^) included three randomly established quadrats (1 × 1 m^2^). The plant species richness (*R*) and Shannon-Wiener (*H′*) index were calculated (Pan et al., 2014). The soil samples (0–15 cm soil layer) were collected and mixed as a composite soil sample. The soil samples were sieved (2 mm), divided into two parts, and transferred at 4°C. One part was for DNA extraction, and the other part was for soil physicochemical analyses. The ring sampler weighing method was used to measure the soil bulk density (BD). The soil water content (SWC) was determined by the gravimetric method. Soil pH was determined using a glass electrode meter (InsMark^™^ IS126, Shanghai, China) in a 1:2.5 soil:water (w/v) mixture. The soil organic carbon (SOC) and soil total nitrogen (TN) were determined by potassium dichromate oxidation and the Kjeldahl method, respectively. The available nitrogen (AN) was measured by the alkali-diffusion method. Soil determination of total phosphorus (TP) and available phosphorus (AP) was performed by alkali fusion-Mo-Sb anti-spectrophotometric and sodium hydrogen carbonate solution-Mo-Sb anti-spectrophotometric methods, respectively. The total potassium (TK) was determined by alkali fusion-atomic absorption spectrophotometry methods. The available potassium (AK) was measured by the acid fusion-atomic absorption spectrophotometry method.

### DNA extraction and PCR amplification

Total DNA was extracted using the CTAB method in accordance with the instructions. The concentration and purity of DNA were examined with 1% agarose gel. The primer set of 515F (5’-GTGCCAGCMGCCGCGGTAA-3′) and 806R (5’-GGACTACHVGGGTWTCTAAT-3′) targeting the bacterial 16S rRNA V4 region and ITS3F (5’-GCATCGATGAAGAACGCAGC) and ITS4R (5’-TCCTCCGCTTATTGATATGC) targeting the fungal IST2 region were used for bacterial and fungal genes sequencing, respectively (Caporaso et al., 2012; Jamil et al., 2020). The bacterial and fungal genes were amplified on Phusion^®^ High-Fidelity PCR Master Mix (New England Biolabs). All the raw sequence reads were conducted using an Illumina NovaSeq 6,000 PE250 platform (Illumina, San Diego, CA, United States). All sequence data are deposited on the NCBI and accessible *via* BioProject IDs of PRJNA851199 for 16S sequences and PRJNA861802 for ITS sequences.

### Data processing and bioinformatics analysis

The QIIME2 system was used for raw data FASTQ files filtered and analyzed (Vazquez-Baeza et al., 2013; Bokulich et al., 2018). The demultiplexed sequences were quality filtered, trimmed, denoised, and merged, and then the chimeric sequences were obtained. After identification and removal by the QIIME2 dada2 plugin, the bacterial ASVs were obtained. Vsearch (2.15.1) software was used to identify the optimized sequences with 95% similarity into fungal OTUs. The representative bacterial sequences were taxonomically classified by alignment against the GREENGENES database. The fungal sequences were taxonomically classified by alignment against the UNITE database (Nilsson et al., 2019).

A Venn diagram was drawn using the R (v 4.1.2) package “plotrix.” The Sankey diagram was generated using the JShare online platform.^1^ The microbial diversity index (Shannon-Wiener and Chao1) was calculated by a core-diversity plugin of QIIME2. The analysis of similarities (ANOSIM) and nonmetric multidimensional scaling (NMDS) were performed using the R (v 4.1.2) package “vegan” (Dixon, 2003). The construction of the molecular ecological networks (MEN) of bacterial and fungal communities was based on the principle of the Molecular Ecological Network Analyses Pipeline (Zhou et al., 2010; Deng et al., 2012). Random matrix theory (RMT) was used to determine the appropriate similarity threshold (St) of molecular ecological networks (Deng et al., 2012). The calculation order follows the rules of decreasing the cutoff from the top, and scan speed refers to the method of regress Poisson distribution only. Cytoscape software (v 3.9.0) was used to visualize the co-occurrence microbial network. The within-module connectivity (Zi) and among-module connectivity (Pi) represent the ecological attributes of the network nodes (Rottjers and Faust, 2018). The network node topologies were classified as peripherals (Zi < 2.5 and Pi <0.62), connectors (Zi < 2.5 and Pi ≥0.62), module hubs (Zi ≥ 2.5 and Pi ≤0.62), and network hubs (Zi ≥ 2.5 and Pi ≥0.62). The connectors, module hubs, and network hubs acted as keystone taxa in the co-occurrence microbial network (He et al., 2017). To determine the ecological processes, phylogenetic and null model analyses were performed (Stegen et al., 2012, 2013). The phylogenic turnover across soil samples was measured by the weighted β-nearest taxon index (βNTI) using the R (v 4.1.2) package “picante” (Kembel et al., 2010). Ecological processes were divided into deterministic processes with |βNTI| values above 2 and stochastic processes with |βNTI| values below 2. If βNTI >2, variable selection plays crucial roles in shaping microbial communities. If βNTI < −2, homogeneous selection is the key assembly process in the microbial community. To further discern the stochastic processes, the Raup–Crick matrix (RCbray) was analyzed *via* the “vegan” package. The conditions of RCbray >0.95, |RCbray| < 0.95, and RCbray < −0.95 indicated drift, undominated processes, and homogenizing dispersal, respectively. Redundancy analysis (RDA) of bacterial or fungal communities and soil and plant properties was conducted in the R (v 4.1.2) package “vegan.” The selection principle of the RDA or CCA model was based on the results of DCA analysis; if the length was >4.0, the CCA model was chosen. Otherwise, the RDA model should be selected. In this study, the value of bacterial sample data was <4.0; thus, RDA was selected. A fungal sample date >4.0 was chosen; thus, CCA was chosen. The Mantel test was used to discern correlations among the bacterial and fungal communities and soil and plant properties based on the Spearman correlation coefficient, and was performed using the R (v 4.1.2) package “vegan.” The data were assessed for normality and homogeneity of variances, and ANOVAs were performed at the 95% confidence level. The ANOVAs were carried out using SPSS (v10.0).

## Results

### Soil and plant characteristics of the five karst tiankengs

Except for AK and AP, the physicochemical parameters of soils varied significantly between different tiankengs (Table 1). The SWC, TN, and AN were significantly higher in XTK. The SOC content was significantly higher in SJXT. The soil TK content ranged from 3.50 to 5.54. All tiankeng soils were slightly acidic, with pH values ranging from 6.19 to 6.67. The plant diversity of karst tiankengs was indicated by Shannon-Wiener (*H*) and richness (*R*) indices and differed among the five karst tiankengs (Supplementary Table 2). The plant species diversity was significantly higher in WJXT (*p* < 0.05).

**Table 1:** The soil properties of the five karst tiankengs.

	BJXT	SJXT	SXT	WJXT	XTK
BD	1.07 ± 0.27a	0.72 ± 0.10b	0.96 ± 0.20ab	1.05 ± 0.28a	0.76 ± 0.24b
SWC	0.34 ± 0.05b	0.28 ± 0.08b	0.28 ± 0.05b	0.20 ± 0.07c	0.50 ± 0.09a
SOC (g kg^−1^)	38.48 ± 30.65bc	66.24 ± 26.38a	43.49 ± 17.95ab	17.99 ± 9.71c	57.54 ± 22.01ab
TN (g kg^−1^)	3.00 ± 1.96b	4.16 ± 1.67ab	3.17 ± 1.15ab	0.87 ± 0.50c	4.76 ± 1.79a
TP (mg kg^−1^)	850.10 ± 383.33a	540.27 ± 105.81b	630.00 ± 128.50ab	218.38 ± 76.01c	835.11 ± 399.44a
TK (g kg^−1^)	4.05 ± 1.24ab	3.50 ± 1.02b	5.54 ± 1.76a	5.40 ± 1.99a	3.69 ± 1.00b
AK (mg kg^−1^)	172.61 ± 65.49a	162.71 ± 44.42a	169.79 ± 28.75a	145.48 ± 46.58a	178.75 ± 48.84a
AP (mg kg^−1^)	1.06 ± 0.17a	1.04 ± 0.03a	1.01 ± 0.02a	1.05 ± 0.05a	1.09 ± 0.06a
AN (mg kg^−1^)	193.31 ± 109.78b	306.32 ± 91.28a	201.75 ± 78.38b	172.01 ± 109.96b	352.27 ± 59.05a
pH	6.19 ± 0.24b	6.19 ± 0.44b	6.36 ± 0.23b	6.36 ± 0.44b	6.76 ± 0.46a

BJXT: Bajiaxiantang, SJXT: Shaojiaxiantang, SXT: Shenxiantang, WJXT: Wangjiaxiantang, XTK: Xiaotiankeng; BD: bulk density, SOC: soil organic carbon, TN: total nitrogen, TP: total phosphorous, TK: total potassium, AK: available potassium, AP: available phosphorous, AN: available nitrogen; Values are mean ± standard error; Different minuscule alphabet means divergence is significant at 0.05 levels.

### Structures of bacterial and fungal communities in karst tiankeng

**Figure 1:**
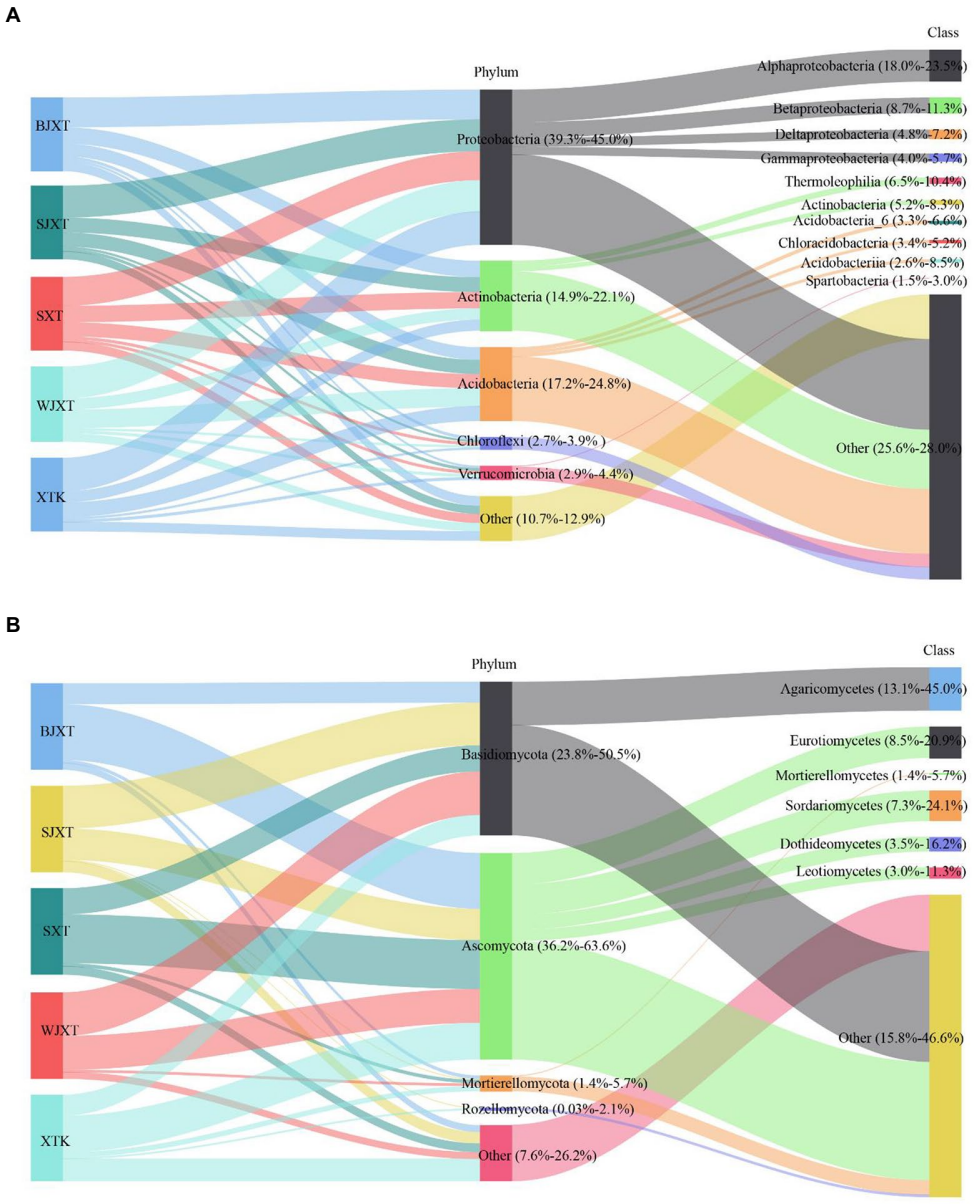
The Sankey diagram of the composition of bacterial **(A)** and fungal **(B)** communities at phylum and class level in five karst tiankengs. BJXT: Bajiaxiantang, SJXT: Shaojiaxiantang, SXT: Shenxiantang, WJXT: Wangjiaxiantang, XTK: Xiaotiankeng.

After quality control, a total of 2,123,723 bacterial clean sequences and 2,508,449 fungal clean sequences were obtained. These sequences were grouped into 19,466 bacterial ASVs and 14,414 fungal OTUs (97% similarity threshold). Only 1,137 bacterial ASVs (5.8% of the total bacterial ASVs) and 195 fungal OTUs (1.4% of the total fungal OTUs) were shared by all karst tiankeng soils (Supplementary Figure 2). At the phylum of bacteria, *Proteobacteria* (39.3%–45.0.97%), *Acidobacteria* (17.2–24.8%), and *Actinobacteria* (14.9–22.1%) were predominant in all karst tiankeng soils (Figure 1A). Proteobacteria were represented by the classes *Alphaproteobacteria* (18.0–23.5%), *Betaproteobacteria* (8.7–11.3%), and *Deltaproteobacteria* (4.8–7.2%). *Actinobacteria* were represented by the classes *Thermoleophilia* (6.5–10.4%) and *Actinobacteria* (5.2–8.3%). In addition, the archaeal phyla *Crenarchaeota* and *Euryarchaeota* were also detected in the karst tiankeng soils. At the phyla of fungal, *Basidiomycota* (23.8–50.5%) and *Ascomycota* (36.2–63.6%) were predominant in all karst tiankeng soils. *Ascomycota* was mainly represented by the classes *Eurotiomycetes* (8.5–20.9%), *Sordariomycetes* (7.3–24.1%), and *Dothideomycetes* (3.5–16.2%; Figure 1B).

**Figure 2:**
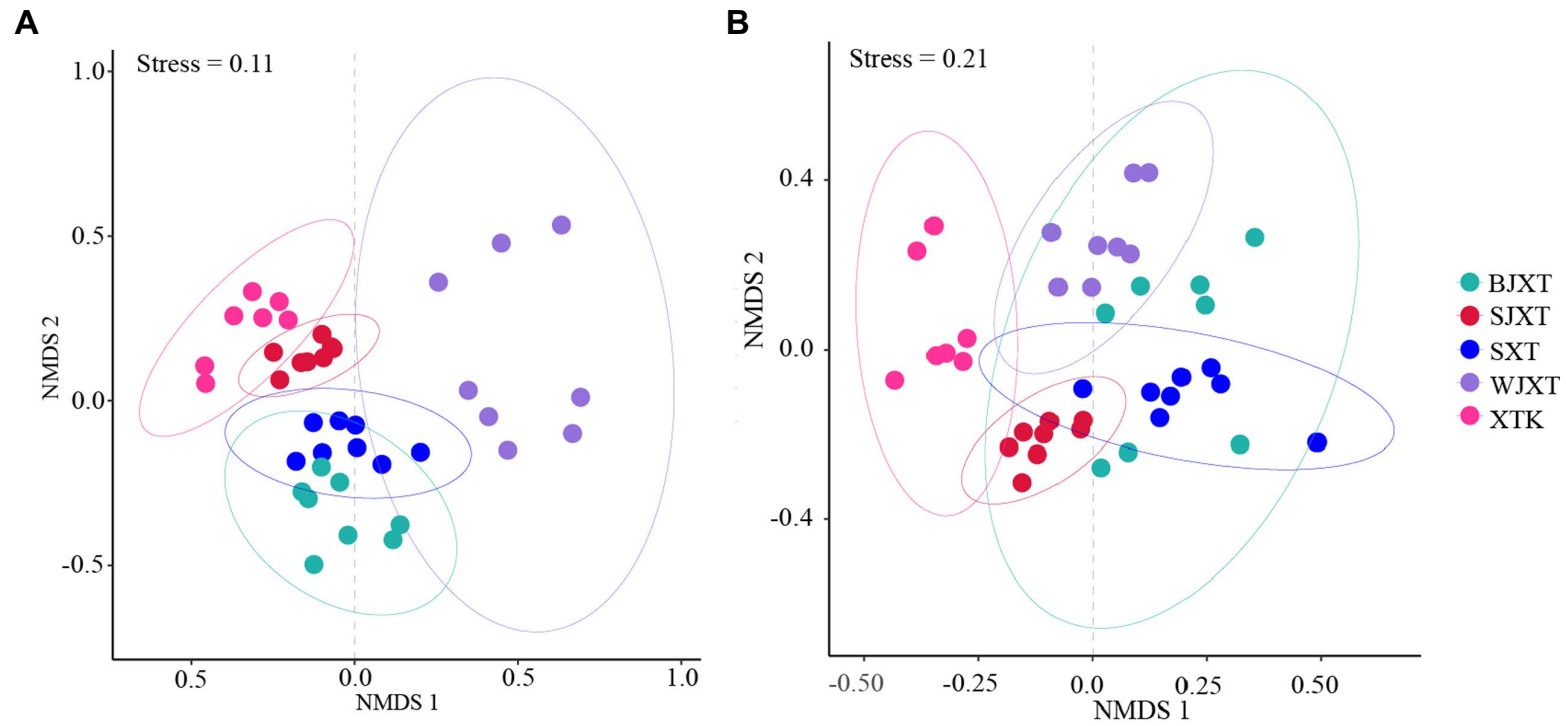
The non-metric multidimensional scaling (NMDS) analysis of bacterial **(A)** and fungal **(B)** communities in five karst tiankengs. BJXT: Bajiaxiantang, SJXT: Shaojiaxiantang, SXT: Shenxiantang, WJXT: Wangjiaxiantang, XTK: Xiaotiankeng.

The alpha diversity of karst tiankeng microbial communities was indicated by the Shannon and Chao1 indices. There was no significant difference in the bacterial community Chao1 index among the five karst tiankengs (*p* = 0.25), whereas a significant difference was observed in the bacterial community Shannon index among the five karst tiankengs (*p* < 0.05). A significant difference was observed in fungal community alpha diversity among the five karst tiankengs (*p* < 0.05; Supplementary Figure 3). The analysis of similarities (ANOSIM; bacterial: *R* = 0.804, *p* = 0.001; fungal: *R* = 0.489, *p* = 0.001) revealed that bacterial and fungal communities from the different karst tiankengs significantly differed from each other (Supplementary Figure 4). The nonmetric multidimensional scaling (NMDS) plot showed that the bacterial communities in BJXT and SXT were closely clustered, while the fungal communities in BJXT, SJXT, SXT, and WJXT were closely clustered and separated from those in XTK (Figure 2).

### Microbial networks and keystone taxa in karst tiankeng

**Figure 3:**
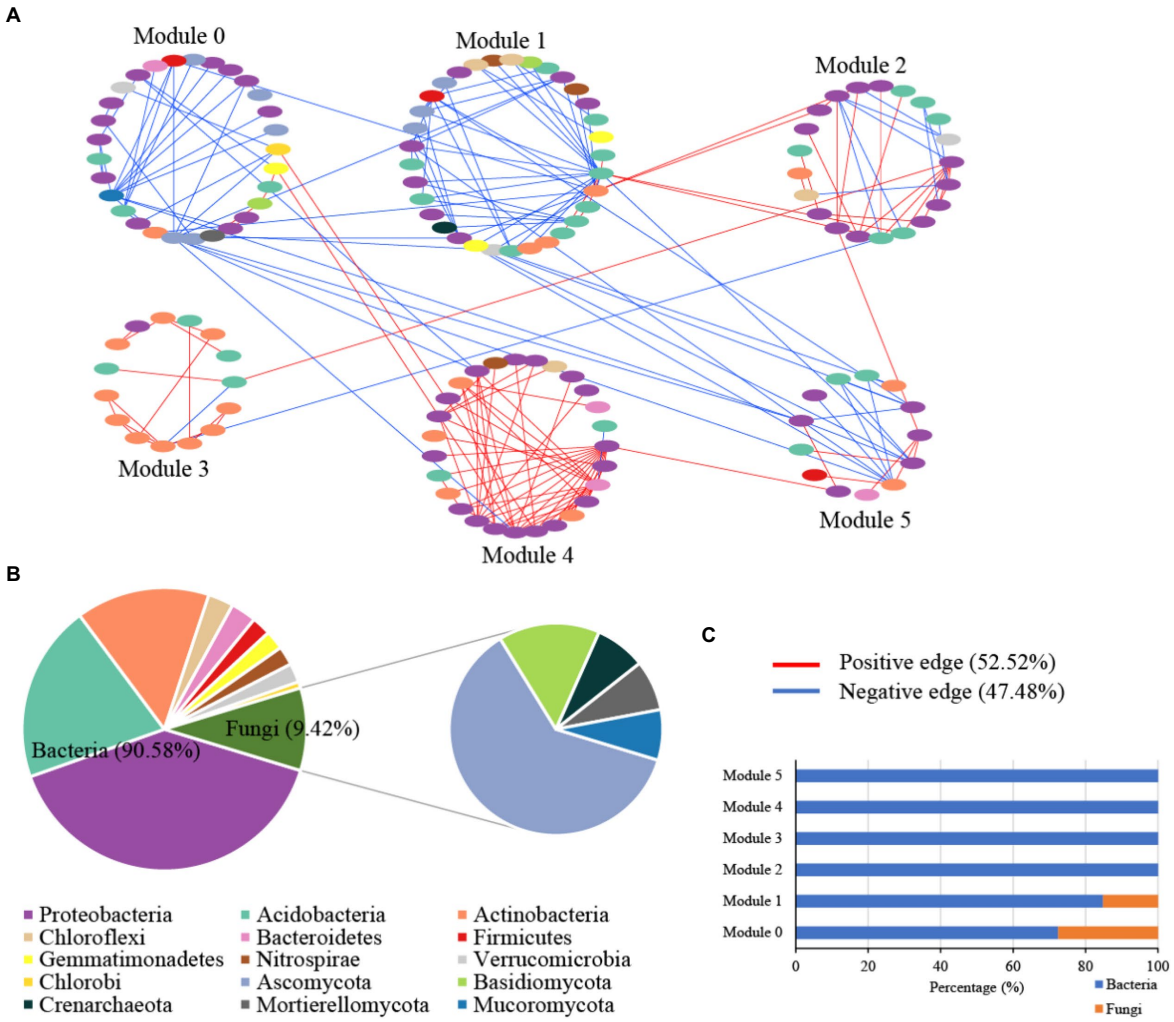
The microbial networks of bacterial and fungal communities of Zhanyi Karst Tiankeng Group by modules **(A)**; The proportion of microbial composition (phylum level) in main module **(B)**; The proportion of bacterial ASVs and fungal OTUs in main module **(C)**.

The microbial random molecular ecological network (MENs) identified the microbe-microbe interactions in karst tiankeng. The karst tiankeng microbial network was a scale-free network (R^2^ = 0.923) and exhibited a good modular structure (modularity >0.4; Table 2). The karst tiankeng microbial network consisted of 185 nodes and 238 edges. More positive interaction edges (52.52%) were observed in the karst tiankeng microbial network. In the network of karst tiankeng soils, most nodes were grouped into six major modules (Figure 3A). Bacteria and fungi accounted for 90.58 and 9.42% of the total nodes, respectively (Figure 3B). The largest modules contain 17.84% of the total nodes. *Proteobacteria* (bacteria), *Acidobacteria* (bacteria), and *Ascomycota* (fungi) dominated in the major modules. Bacteria dominated in all the major modules, and fungi were mainly located in modules 0 and 1 (Figure 3C).

**Table 2:** Topological properties of microbial networks in five karst tiankengs.

	*St*	Nodes	Edges	R^2^ of power-law	*avgCC*	*GD*	*HD*	*avgK*	Density	Modularity
Karst Tiankeng	0.710	185	238	0.923	0.088	5.490	4.182	2.573	0.014	0.729

avgCC: Average clustering coefficient; GD: Average path distance; HD: Harmonic geodesic distance; avgK: Average degree.

**Figure 4:**
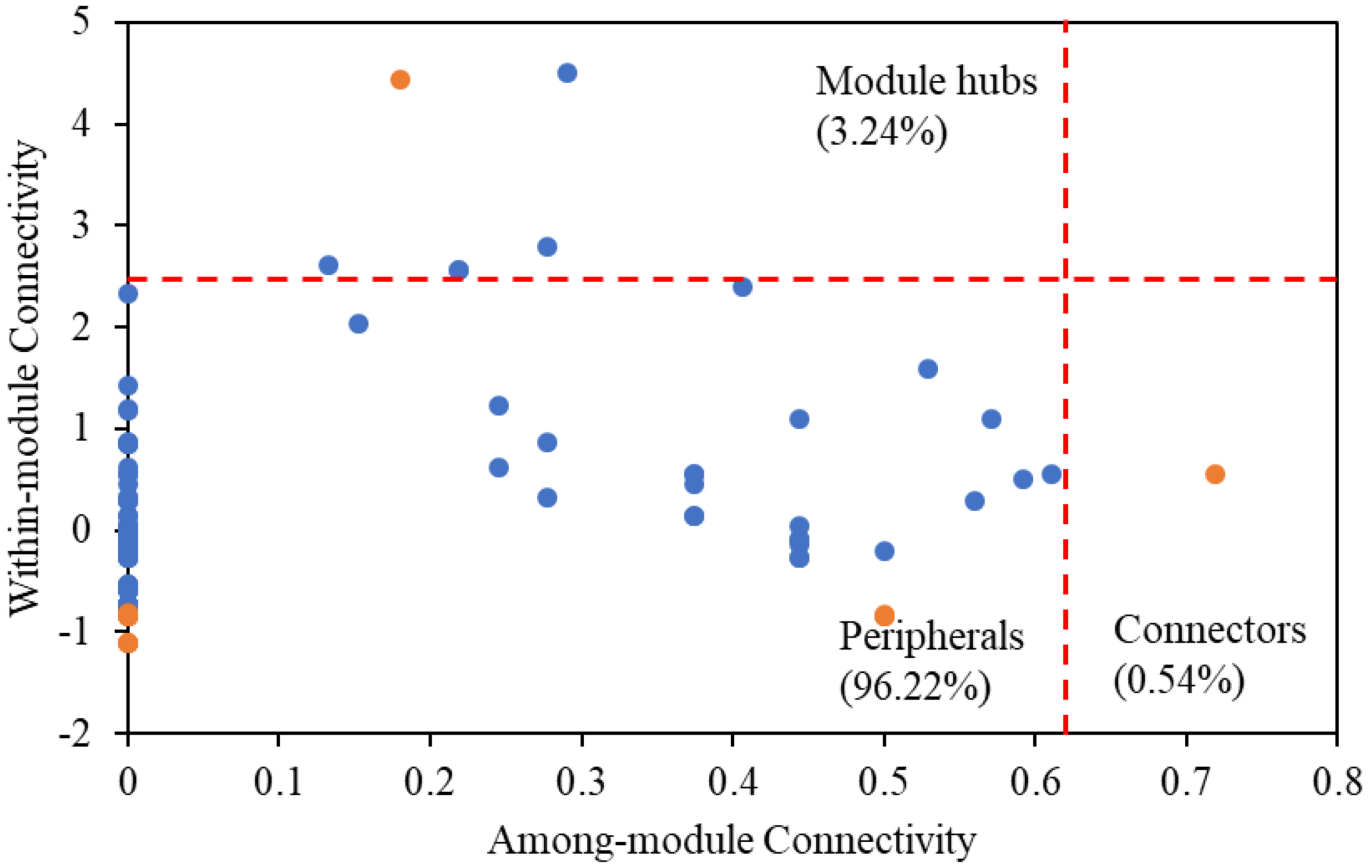
The ZP-plots shows distribution of ASVs/OTUs according to their module-based topological roles in the networks of five karst tiankengs. Each blue or orange dot represents a bacterial ASVs or fungal OTUs, respectively.

Among all nodes, 96.22% belonged to peripherals in karst tiankengs (Figure 4). Seven keystone taxa (five bacteria keystone taxa and two fungi keystone taxa) were identified in the karst tiankeng network, including six module hubs (3.24%) and one connector (0.54%). The dominant keystone taxa were *Proteobacteria* (bacteria) with an abundance of 42.86% of all keystone taxa. Bacteria of *Bradyrhizobium* (genus), *Steroidobacter* (genus), *Micrococcales* (order), *Ellin6513* (order), and fungi of *Umbelopsis* (genus) and *Fusarium* (genus) were the keystone taxa in the karst tiankeng network (Supplementary Table 3). All fungal keystone taxa of the karst tiankeng network were located in module 0.

### Microbial communities assembly in karst tiankeng

**Figure 5:**
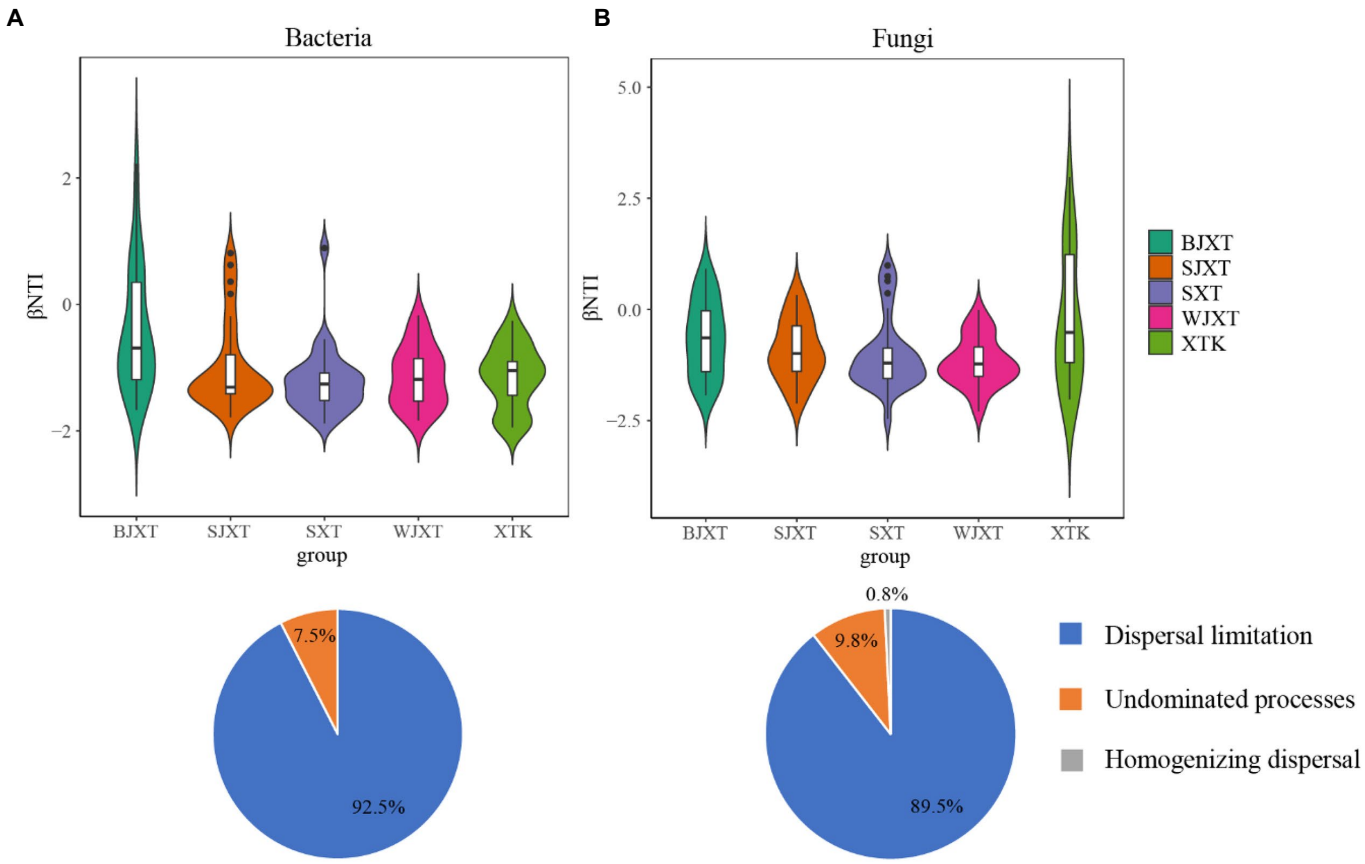
Phylogenetic and null model analyses revealing assembly processes of bacterial **(A)** and fungal **(B)** communities in five karst tiankeng tested by βNTI values, and percentage of each ecological process contributed to the assembly of bacterial and fungal communities in five karst tiankengs.

The median phylogenetic turnover was between −2 and 2, which indicated that stochastic processes controlled the bacterial community assembly. Dispersal limitation was the key process in bacterial community assembly, with a contribution of 92.5%. Similarly, stochastic processes are crucial assembly processes in fungal community composition. Dispersal limitation and undominated processes contributed 89.5 and 9.8%, respectively, to the fungal community assembly (Figure 5).

### Relationships between microbial communities and soil and plant variables

**Figure 6:**
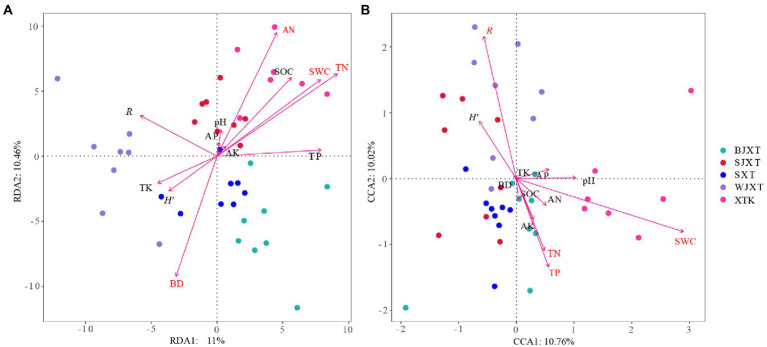
The redundancy analysis (RDA) of the bacterial **(A)** and fungal **(B)** communities with soil and plant properties in five karst tiankengs. Mark the significant factors in red font.

RDA was conducted to investigate the impact of soil properties and plant properties (richness and Shannon-Wiener) on bacterial and fungal communities. Among all the variables investigated, TN, AN, SWC, and BD had significant relationships with the bacterial communities (Figure 6A). SWC, *R*, TP, and TN were significantly associated with fungal communities (Figure 6B). The Mantel test results showed that BD, SWC, TN, TP, AP, and AN had significant relationships with the bacterial communities, while SWC, TN, AN, and *H′* had significant relationships with the fungal communities (*p* < 0.05, Supplementary Table 4). Significant correlations were also observed between the main microbial phylum and soil or plant variables (Supplementary Table 5).

## Discussion

### Characteristics of microbial communities in karst tiankeng

The constant physicochemical and microclimate conditions in the subsurface support a stable ecosystem in karst tiankeng. The bacterial communities in karst tiankeng were dominated by *Proteobacteria* and *Acidobacteria*. Similar results were reported for Shenmu karst tiankeng located in Guangxi, southwestern China (Pu et al., 2019). *Proteobacteria* are considered to play an important role in phylogenetic and ecological values and have highly diverse metabolic capabilities (Kang et al., 2019; von Borzyskowski et al., 2019). *Acidobacteria* species are considered to play a key role in nutrient cycles and perform the function of organic matter decomposition (Eichorst et al., 2018). In addition, the other abundant phyla included *Actinobacteria*, *Chloroflexi*, and *Verrucomicrobia*. *Actinobacteria* are considered the most important source of bioactive compounds, especially commercially available antibiotics (Barka et al., 2016; Rangseekaew and Pathom-aree, 2019). *Actinobacteria* (14.9–22.1%) ranked the third most abundant phyla in the karst tiankeng, which may indicate that *Actinobacteria* in the unique habitat of karst tiankeng are expected to be a good source of bioactive compound discovery. *Chloroflexi* and *Verrucomicrobia* are regarded as having a strong ability to survive in poor nutrient conditions (Pan et al., 2014; Yang et al., 2021), which may help karst tiankeng microbial communities resist external environmental disturbances. The fungal communities in karst tiankeng were dominated by *Ascomycota* and *Basidiomycota*. Previous research has shown that *Ascomycota* and *Basidiomycota* have a strong ability to decompose cellulose, and improve rock weathering in karst habitats (Baldrian et al., 2012; Xiao et al., 2022). Some of the fungal classes observed in our study were also detected in karst caves, such as *Sordariomycetes* and *Dothideomycetes* (Zhang et al., 2017; Man et al., 2018). The underground drainage system of karst landscapes is characterized by karst tiankengs and caves (Legatzki et al., 2011). The intricate hydrological system of the karst system links two unique habitats.

However, the shared bacterial ASVs and fungal OTUs among the five karst tiankengs accounted for less than 10% (Supplementary Table 1), indicating significant differences among bacterial and fungal communities among the five karst tiankengs. Different niches between karst tiankengs drive the evolution of microbial communities and maintain unique microbial populations. The results of the NMSD analysis also confirmed significant differences in bacterial and fungal communities in different karst tiankengs (Figure 2). Karst tiankengs, as isolated habitats, have a distinct island-like effect (Itescu, 2019). The isolation effect of the vertical cliff may vary between different karst tiankengs (Shui et al., 2018), leading to differences in microbial community evolution, composition, and genetic diversity. Affected by geological conditions, the external environment, and isolation effects (Wang et al., 2020), the different habitats between karst tiankengs may maintain diverse microbial communities.

### Microbial co-occurrence network in karst tiankeng

Our study constructed an integrated microbial network by using different samples from five karst tiankengs, which suggests that the karst tiankeng microbial communities have more interactions within the niche (Figure 3). In the unique habitat of karst tiankengs, microbes may adopt different survival strategies (Jiang et al., 2022a). The inner microbial interactions play an important role in community stability and are determined by the basic dynamics of species to promote their survival (Zhou et al., 2011; Banerjee et al., 2018). A higher proportion of positive interaction edges of microbial networks (52.52%) suggested that bacterial and fungal communities form a tight organization through cooperation, thereby enhancing the complexity of the community structure and the stability of the karst tiankeng ecosystem (Wang Y. et al., 2022). Additionally, the collaboration between autotrophic and heterotrophic microorganisms contributes to their growth and metabolism and probably enhances community functions (Nadell et al., 2016). The microbial network showed good modularity, indicating nonrandom patterns of microbial interactions in karst tiankeng (Olesen et al., 2007).

The dominant phyla in the microbial network were *Proteobacteria* (bacteria) and *Ascomycota* (fungi), which are also the main soil microbial groups in various habitats (Sun et al., 2017; Cui et al., 2019). Based on the main functions of these microbial groups, it can be inferred that microbes involved in nutrient cycles and energy metabolism can survive well in karst tiankeng. Keystones are considered to play an important role in maintaining the stability of the microbial community (Mondav et al., 2017). A total of five bacterial keystones and two fungal keystones were identified in the karst tiankeng network (Supplementary Table 2). Previous studies demonstrated that *Bradyrhizobium* bacteria closely interact with plant roots and is abundantly present at high levels of plant diversity (Berg and Smalla, 2009; Xiao et al., 2021). *Bradyrhizobium* serves as a keystone in the network, which may be related to the abundant vegetation of karst tiankeng. As a member of *Proteobacteria*, *Steroidobacter* can access C and nutrients in oligotrophic conditions (Jeewani et al., 2020). The keystones of *Bradyrhizobium* and *Steroidobacter* play a key role in organic matter decomposition and nutrient cycling by connecting other microbial members in the network. In addition, fungal keystones of *Fusarium* are ubiquitous in karst ecosystems (Wang et al., 2016). *Fusarium* can secrete cellulase to decompose carbon, and participate in the dissolution of soil insoluble phosphorus, effectively improving the acquisition of phosphorus by plants (Yang et al., 2018). In general, keystones play a key role in maintaining the ecological function of the karst tiankeng ecosystem.

### Community assembly processes and the impact of environmental variables on microbial communities

The assembly of bacterial and fungal communities is mainly a stochastic process, with the dominance of dispersal limitation in five karst tiankengs, indicating the spatial heterogeneity of karst tiankengs. The occurrence of dispersal limitation in the karst tiankeng indicates a weakening selection, which may result from the barrier of the vertical cliff. Most previous studies suggested that deterministic processes have a greater impact on the formation of bacterial communities (Cheng et al., 2021; Han et al., 2022; Wang H. et al., 2022). Our results may be explained by the complex environment in the karst tiankeng. The abundant vegetation cover of karst tiankeng leads to a large accumulation of vegetation litter, which enriches soil nutrients and ultimately promotes the abundance of bacterial communities (Liu et al., 2021). The nutrient-rich soil environment allows bacterial communities can thrive in the unique habitat of karst tiankengs. Additionally, bacterial communities can be transferred through the karst hydrological system (Mulec et al., 2019). These ecological processes were mixed to increase the stochastic process of bacterial community assembly in karst tiankeng. Deterministic processes were usually dominant in habitats with steep environmental gradients (Huang et al., 2022). Although the environments of these five karst tiankengs exhibit differences, it does not meet the characteristics of a steep environmental gradient. On the contrary, the interior of karst sinkholes maintains a relatively stable environment due to the effect of pit wall isolation. Thus, stochastic processes play a leading role in the construction of fungal communities in karst tiankengs. The dispersal limitation of fungal communities was consistent with previous studies (Li et al., 2020). The spread of fungal spores is usually limited to short distances, and the unique topography of karst tiankengs significantly reduces the spread of fungal spores. It is important to note that stochastic processes tend to occur before stable microbial communities form; our results may reflect that bacterial and fungal communities in karst tiankengs are still in an unstable state.

The soil and plant properties of the five karst tiankengs showed high habitat heterogeneity. Environmental variables are closely related to microbial diversity and community composition (Gao et al., 2017; Song et al., 2018). The RDA and mental test results showed that soil and plant properties were significantly associated with bacterial and fungal communities of the five karst tiankengs (Figure 6; Supplementary Table 1). SWC has been widely demonstrated to construct soil bacterial and fungal communities (Kaisermann et al., 2015; Long et al., 2021; Lin et al., 2022). Soil moisture content can directly affect the survival activity of microbes, and affect microbial growth by indirectly regulating the distribution of soil nutrients (Clark et al., 2009; Manzoni et al., 2014). For fungal communities, the soil water may help the expansion of soil hyphal networks (Hawkes et al., 2011). Our previous studies also indicated that the abundant soil water content in the karst tiankeng may make it easy for microorganisms to colonize (Jiang et al., 2022a). High SOC contents in karst regions, and soil nitrogen contents are more critical for bacterial communities (e.g., *Proteobacteria*; Li et al., 2022). Previous studies have indicated that soil structure with different bulk densities affects the spread and activity of bacteria in soil (Juyal et al., 2021). The high heterogeneity of karst habitats may affect bacterial communities by altering soil bulk density. Karst areas are generally limited by phosphorus (Chen et al., 2018a) and thus affect microbial communities. Liu et al. (2012) studies also reported that soil TP content is the limiting factor of microbial growth. Yang et al. (2017) study found that soil phosphorus has a more important effect on soil fungal communities than soil carbon-nitrogen ratios, which is consistent with our results. Fungal communities have a close relationship with plant diversity (He et al., 2008). Fungal communities are a key player in breaking down plant litter (Voriskova and Baldrian, 2013). The abundant plants in the karst tiankeng underground forest provide a high-quality environment for fungal communities.

## Conclusion

This study provides a comprehensive assessment of the bacterial and fungal communities in karst tiankeng. The dominant phyla in the five karst tiankengs were mainly included *Proteobacteria*, *Acidobacteria* (bacterial), *Ascomycota,* and *Basidiomycota* (fungal). The diversity and composition of bacterial and fungal communities significantly differed among the five karst tiankengs. The co-occurrence network indicated that microorganisms preferred to survive in karst tiankengs in a modular manner, and abundant taxa relied more on partnerships to adapt to the environment. The keystones might play a critical role in nutrient cycles and energy metabolism. The bacterial communities were mainly related to SWC, TN, AN, and BD, while the fungal communities were mainly related to SWC, TP, and plant diversity. These observations provide improved knowledge about the structure, composition, community assembly processes, and co-occurrence patterns of bacterial and fungal communities in karst tiankeng ecosystems. This study contributes to further studies of the diversity and interaction patterns of bacterial and fungal communities in karst tiankeng worldwide and explores the value of the biodiversity conservation pool of karst tiankeng.

## Data availability statement

The datasets presented in this study can be found in online repositories. The names of the repository/repositories and accession number(s) can be found in the article/supplementary material.

## Author contributions

CJ and WS designed the study. CJ, XS, YL, KW, and HL carried out field sampling and plot surveys. CJ performed the experiments, analyzed the data, and wrote the manuscript. SZ provided technical support to this study. WS provided critical comments and edited the manuscript. All authors contributed to the article and approved the submitted version.

## Funding

This work was supported by the National Natural Science Foundation of China (41871198).

## Conflict of interest

The authors declare that the research was conducted in the absence of any commercial or financial relationships that could be construed as a potential conflict of interest.

## Publisher’s note

All claims expressed in this article are solely those of the authors and do not necessarily represent those of their affiliated organizations, or those of the publisher, the editors and the reviewers. Any product that may be evaluated in this article, or claim that may be made by its manufacturer, is not guaranteed or endorsed by the publisher.

## Supplementary material

The Supplementary material for this article can be found online at: https://www.frontiersin.org/articles/10.3389/fmicb.2022.1002198/full#supplementary-material

## Footnotes

^1^ https://jshare.com.cn/demos/eYEsqk
