# Supplementary material for: The characterization of microbial communities and associations in karst tiankeng
Source: Front Microbiol. 2022 Oct 19;13:1002198. doi: 10.3389/fmicb.2022.1002198 (PMC9632645; doi:10.3389/fmicb.2022.1002198)
Supplement: Supplementary file 1 [file Data_Sheet_1.pdf]

Supplementary Material

Supplementary Table S1. The morphological characteristics of the five karst tiankengs.

	BJXT	SJXT	SXT	WJXT	XTK
Length (m)	240	277	422	113	80
Width (m)	198	248	349	92	63
Depth (m)	70	76	149	122	78
Number of vertical cliffs	2	1	2	1	4

BJXT: Bajiaxiantang, SJXT: Shaojiaxiantang, SXT: Shenxiantang, WJXT: Wangjiaxiantang, XTK: Xiaotiankeng.

Supplementary Table S2. The vegetation communities feature of the five karst tiankengs.

	Species richness (*R*)	Shannon-Wiener (*H'*)	Main species
BJXT	16.25±3.33c	2.11±0.35ab	*Quercus variabilis, Alangium chinense, Cyclobalanopsis glauca*
SJXT	23.13±3.76ab	2.32±0.25ab	*Quercus glauca Thunb., Myrsine africana* Linn., *Pyracantha fortuneana* (Maxim.) Li
SXT	18.75±3.85bc	2.15±0.31bc	*Pistacia weinmannifolia* J. Poisson ex Franch., *Quercus guyavifolia*, *Myrsine africana* Linn.
WJXT	25.88±5.89a	2.56±0.27a	*Swida oblonga, Cyclobalanopsis glauca*
XTK	18.57±3.26bc	1.98±0.30c	*Trachycarpus fortunei* (Hook.) H. Wendl. *Carpinus monbeigiana* Hand.-Mazz.

BJXT: Bajiaxiantang, SJXT: Shaojiaxiantang, SXT: Shenxiantang, WJXT: Wangjiaxiantang, XTK: Xiaotiankeng; Values are mean ±standard error; Different minuscule alphabet means divergence is significant at 0.05 levels.

Supplementary Table S3. The details of keystone taxa in microbial network.

	**ID**	**No. module**	**Pi**	**Zi**
Module hubs	k__Bacteria;p__Proteobacteria;c__Alphaproteobacteria;o__Rhizobiales;f__Bradyrhizobiaceae;g__Bradyrhizobium	2	0.219	2.567
	k__Bacteria;p__Proteobacteria;c__Gammaproteobacteria;o__Xanthomonadales;f__Sinobacteraceae;g__Steroidobacter;s__	2	0.219	2.567
	k__Bacteria;p__Actinobacteria;c__Actinobacteria;o__Micrococcales;f__;g__;s__	3	0.278	2.799
	k__Bacteria;p__Acidobacteria;c__DA052;o__Ellin6513;f__;g__;s__	1	0.290	4.504
	k__Bacteria;p__Proteobacteria;c__Gammaproteobacteria;o__Xanthomonadales;f__Sinobacteraceae;g__Steroidobacter;s__	4	0.133	2.611
	k__Fungi;p__Mucoromycota;c__Umbelopsidomycetes;o__Umbelopsidales;f__Umbelopsidaceae;g__Umbelopsis	0	0.180	4.434
Connectors	k__Fungi;p__Ascomycota;c__Sordariomycetes;o__Hypocreales;f__Nectriaceae;g__Fusarium	0	0.719	0.557

Supplementary Table S4 Mantel test results that were used to discern correlations among the bacterial and fungal communities UniFrac distances and soil or vegetation properties. The values shown are correlation coefficients based on the Spearman method.

	Bacterial communities		Fungal communities	
	r	*p*	*r*	*p*
BD	0.143	**0.029**	0.131	0.052
SWC	0.218	**0.005**	0.180	**0.021**
SOC	0.105	0.071	0.091	0.125
TN	0.270	**7e-04**	0.168	**0.017**
TP	0.329	**5e-04**	0.118	0.103
TK	0.008	0.418	-0.064	0.757
AK	0.025	0.345	0.022	0.391
AP	0.154	**0.035**	0.070	0.208
AN	0.198	**3e-04**	0.094	**0.025**
pH	0.130	0.056	0.051	0.284
R	0.096	0.090	0.108	0.086
H	0.093	0.099	0.171	**0.020**

Supplementary Table S5 The Significant correlations based on the Pearson correlation coefficient between main microbial phylum and soil or plant variables.

	**BD**	**SWC**	**SOC**	**TN**	**TP**	**TK**	**AK**	**AP**	**AN**	**pH**	**R**	**H**
Proteobacteria	-0.31	0.29	0.10	0.08	0.16	-0.13	0.14	0.22	0.22	0.24	0.12	0.00
Actinobacteria	0.07	-0.15	0.17	0.19	0.23	-0.11	-0.07	-0.14	-0.18	-0.23	**-0.34***	-0.03
Acidobacteria	0.13	-0.19	-0.25	**-0.32***	**-0.46****	0.19	-0.07	0.03	-0.05	-0.01	**0.38***	0.15
Chloroflexi	0.10	0.25	0.08	0.18	0.24	0.18	0.26	-0.05	0.06	-0.08	**-0.37***	-0.24
Verrucomicrobia	-0.03	-0.24	-0.07	-0.19	**-0.32***	0.23	-0.02	-0.11	-0.05	0.07	**0.31***	-0.02
Bacteroidetes	-0.22	**0.45***	0.14	0.23	0.29	-0.30	0.01	-0.16	0.28	**0.33***	-0.06	-0.03
Gemmatimonadetes	0.26	0.01	-0.08	0.08	0.31	-0.07	-0.10	-0.19	-0.06	0.01	**-0.47****	-0.07
Planctomycetes	0.15	-0.18	-0.17	-0.15	-0.25	0.19	-0.03	-0.02	0.00	-0.17	0.08	-0.02
Nitrospirae	-0.25	**0.60*****	**0.41****	**0.58*****	**0.35***	**-0.35***	0.10	0.11	**0.42****	0.20	**-0.33***	**-0.40***
Firmicutes	0.07	-0.06	-0.10	-0.09	0.26	-0.08	0.02	-0.05	-0.21	-0.14	-0.20	-0.05

Basidiomycota	-0.27	-0.25	0.17	-0.01	-0.21	-0.07	-0.23	0.02	0.11	**-0.35***	0.17	0.05
Ascomycota	**0.41****	-0.12	**-0.33***	-0.17	0.13	0.15	0.08	-0.13	**-0.34***	0.14	-0.18	0.15
Mortierellomycota	-0.08	**0.34***	0.10	0.14	**0.35***	0.23	0.23	0.21	0.14	0.05	-0.26	-0.11
Rozellomycota	-0.27	0.35*	0.23	0.30	0.20	-0.18	0.03	0.06	0.32	0.20	-0.14	**-0.34***
unclassified	0.05	**0.41****	0.00	0.08	0.10	0.05	0.03	0.16	0.18	0.14	-0.06	-0.23
Mucoromycota	0.07	**-0.32***	-0.25	-0.31	-0.29	-0.12	-0.19	-0.12	-0.28	0.25	**0.59*****	**0.45****
Chytridiomycota	0.08	0.05	0.02	0.21	0.08	0.00	-0.03	-0.10	0.01	-0.06	-0.29	-0.14
Anthophyta	0.10	**0.51*****	0.01	0.13	0.26	0.02	**0.34***	0.09	0.05	0.23	-0.22	-0.26
Chlorophyta	0.22	0.03	-0.27	-0.16	0.04	0.13	**0.35***	-0.22	-0.16	0.14	-0.05	0.12
Nematoda	0.04	**0.36***	-0.08	0.03	**0.35***	0.06	0.03	-0.02	0.19	**0.47****	-0.12	-0.12

* indicates $P < 0.05$; ** indicates $P < 0.01$, *** indicates $P < 0.001$.

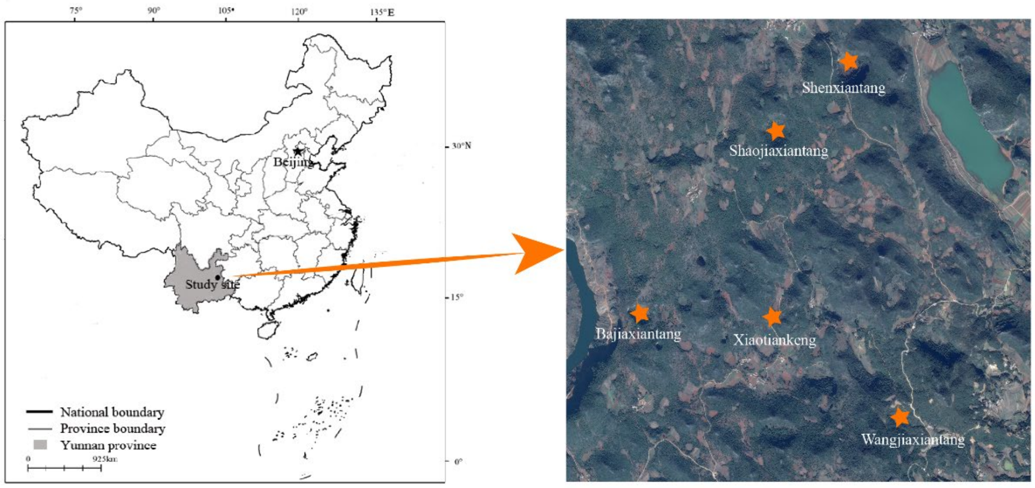

Supplementary Figure 1. Location of study site on the map of China and sampling sites at Zhanyi tiankeng group.

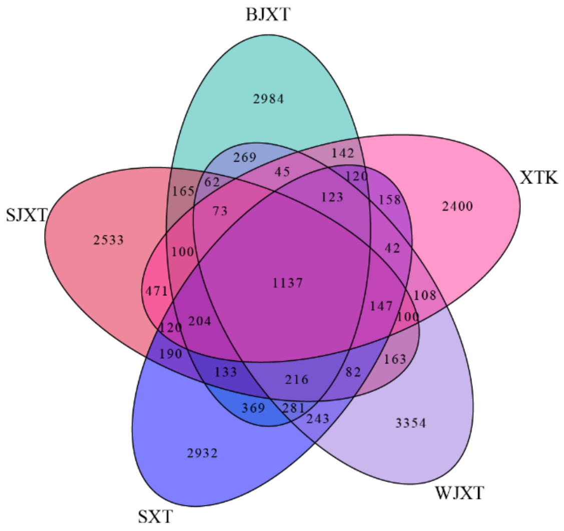

Supplementary Figure 2. The Veen diagram of the exclusive and shared ASV found among the different karst tiankeng.

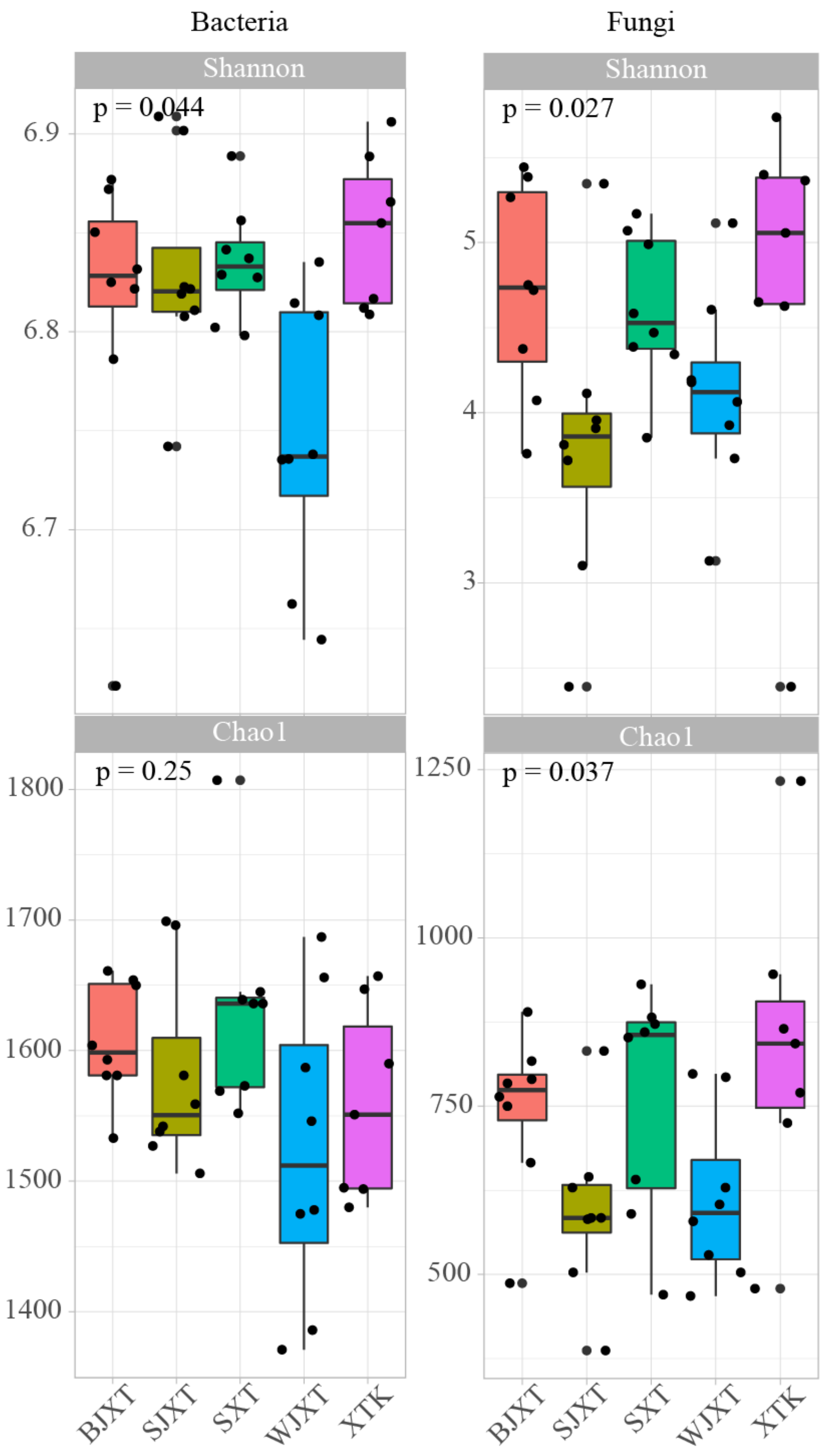

Supplementary Figure 3. The alpha diversity of bacterial and fungal communities in five karst tiankengs.

.

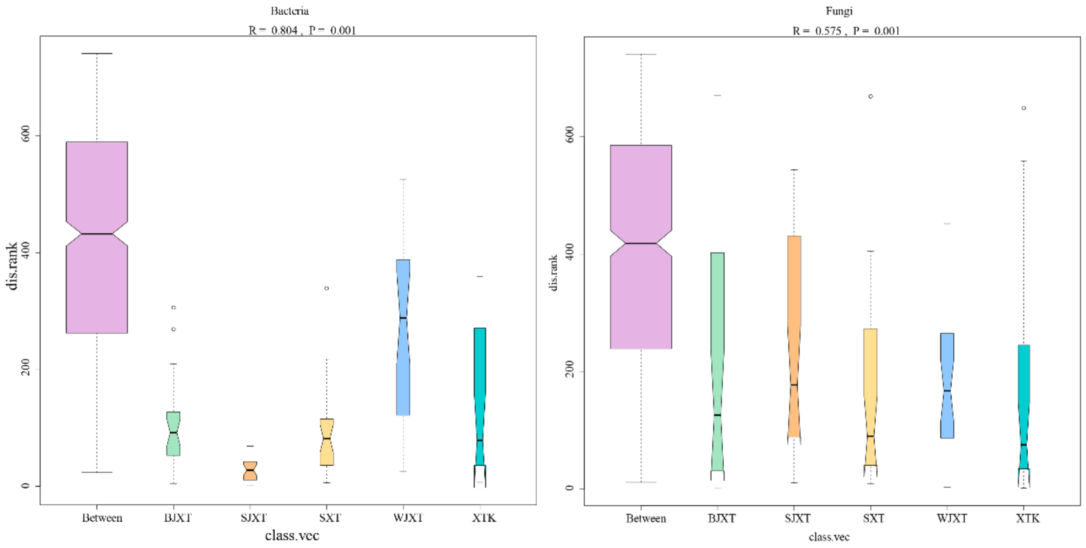

Supplementary Figure 4. The analysis of similarities (AMOSIM) of bacteria and fungal communities composition in five karst tiankengs.
